# Keutel Syndrome, a Review of 50 Years of Literature

**DOI:** 10.3389/fcell.2021.642136

**Published:** 2021-04-29

**Authors:** M. Leonor Cancela, Vincent Laizé, Natércia Conceição, Hervé Kempf, Monzur Murshed

**Affiliations:** ^1^Centre of Marine Sciences (CCMAR), University of Algarve, Faro, Portugal; ^2^Faculty of Medicine and Biomedical Sciences, University of Algarve, Faro, Portugal; ^3^Algarve Biomedical Center, University of Algarve, Faro, Portugal; ^4^UMR 7365 CNRS-Université de Lorraine, IMoPA, Vandoeuvre-lès-Nancy, France; ^5^Department of Medicine and Faculty of Dentistry, McGill University, Montreal, QC, Canada; ^6^Shriners Hospital for Children, Montreal, QC, Canada

**Keywords:** recessive genetic disorder, matrix Gla protein, abnormal calcification, cartilaginous tissues, mouse model, gamma-carboxylation, phosphorylation

## Abstract

Keutel syndrome (KS) is a rare autosomal recessive genetic disorder that was first identified in the beginning of the 1970s and nearly 30 years later attributed to loss-of-function mutations in the gene coding for the matrix Gla protein (MGP). Patients with KS are usually diagnosed during childhood (early onset of the disease), and the major traits include abnormal calcification of cartilaginous tissues resulting in or associated with malformations of skeletal tissues (e.g., midface hypoplasia and brachytelephalangism) and cardiovascular defects (e.g., congenital heart defect, peripheral pulmonary artery stenosis, and, in some cases, arterial calcification), and also hearing loss and mild developmental delay. While studies on *Mgp*^–/–^ mouse, a faithful model of KS, show that pathologic mineral deposition (ectopic calcification) in cartilaginous and vascular tissues is the primary cause underlying many of these abnormalities, the mechanisms explaining how MGP prevents abnormal calcification remain poorly understood. This has negative implication for the development of a cure for KS. Indeed, at present, only symptomatic treatments are available to treat hypertension and respiratory complications occurring in the KS patients. In this review, we summarize the results published in the last 50 years on Keutel syndrome and present the current status of the knowledge on this rare pathology.

## Introduction

**FIGURE 1:**
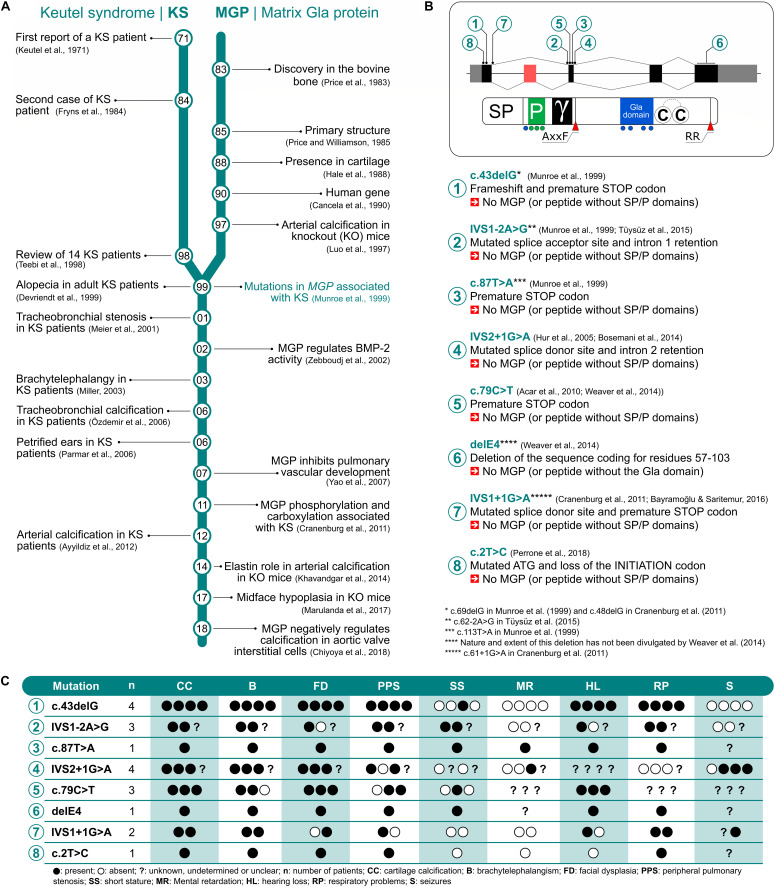
Literature timeline for Keutel syndrome (KS) and matrix Gla protein (MGP) **(A)**. *MGP* gene and protein structure mapping the eight mutations associated with KS **(B)**. Most common traits observed in KS patients genotyped for *MGP* **(C)**.

Keutel syndrome (KS; OMIM #245150; ORPHANET #85202) was first described in 1971 in two consanguineous siblings (Keutel et al., 1971; Figure 1A). It is an extremely rare autosomal recessive disorder (estimated prevalence of 1 in 1,000,000), which is usually diagnosed during childhood, although it can remain undetected until adulthood. Forty-two cases have been reported so far, with half in Turkish population (Table 1). However, this number may be underestimated as the main clinical characteristics of KS – midface hypoplasia, abnormal cartilage calcification, brachytelephalangism, peripheral pulmonary stenosis – are also observed in other disorders (for example, chondrodysplasia punctata or Conradi’s disease), leading to the misdiagnosis of some forms of KS into another disease (Say et al., 1973). Moreover, reports of mild cases of KS and intrafamilial phenotypic variability (Tüysüz et al., 2015; cases 36 and 37 in Table 1) indicate that some patients may have undetectable forms of the disease. Although parental consanguinity is prevalent in KS, pregnancy is normal (except for one reported case of repeated miscarriages) and blood parameters, including calcium and phosphate serum levels, are usually unremarkable. The prognosis of KS is good in most patients and their life expectancy mostly depends on the severity of the pulmonary complications. In 1999, almost 30 years after its first description, the pathogenesis of KS was associated with a non-functional matrix Gla protein (Munroe et al., 1999; Figure 1A), an extracellular matrix (ECM) protein known to function as an inhibitor of tissue calcification (Luo et al., 1997). This discovery allowed a better understanding of the mechanisms underlying this disease and identified a target for a possible therapeutic solution, although much remains to be done.

**TABLE 1:** Clinical characteristics of Keutel syndrome.

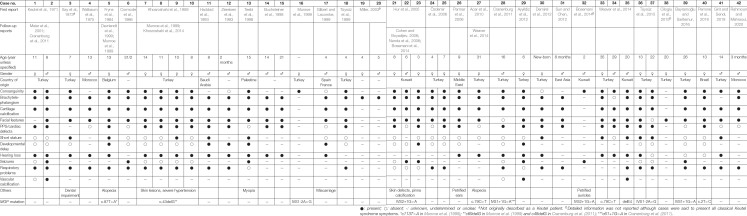

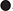
: *present; 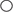: absent; -: unknown, undetermined or unclear. ^*a*^Not originally described as a Keutel patient. ^*b*^Detailed information was not reported although cases were said to present all classical Keutel syndrome symptoms. *c113T>A in Munroe et al. (1999); **c69delG in Munroe et al. (1999) and c48delG in Cranenburg et al. (2011); ***c61+1G>A in Cranenburg et al. (2011).*

In this short review that marks the 50 years of the discovery of Keutel syndrome, we will describe the main clinical characteristics of KS, give an overview on the mutations identified in *MGP* gene and present data collected from knockout and transgenic mouse models that help us to understand KS-associated pathologies.

## Description of the Disease

The clinical traits of KS are very diverse (Table 1), and each is highly variable in its severity and occurrence. Yet, the most consistent traits in this disorder are: (i) dysmorphic facial abnormalities including sloping forehead, midface hypoplasia, depressed nasal bridge and hypoplastic alae nasi, receding chin and sometimes short palate; (ii) abnormal cartilage calcification in the larynx, the respiratory tract, the ears, the nose and the ribs; and (iii) brachytelephalangism that, in around 75% of the cases, is characterized by shortening and broadening of the first to fourth distal phalanges (punctate epiphyses), with sparing of the fifth phalange, although different combinations have been found sporadically in some patients and without systematic bilaterality (Miller, 2003). Additional symptoms found in almost all patients include multiple peripheral pulmonary artery stenoses, mild to severe unilateral or bilateral sensorineural, mixed hearing loss, and intellectual disability that ranges from borderline intelligence to mild developmental delay, short stature, and respiratory conditions including dyspnea, wheezing, cough, and infections (Table 1). Other inconsistent symptoms are also observed in isolated individuals affected by KS such as seizures or epileptic events (Teebi et al., 1998; Hur et al., 2005), otitis media and/or sinusitis, dental malocclusion, and cardiovascular defects such as ventricular septum defects and ventricular hypertrophy (Table 1).

Long-term follow-up studies revealed additional features in aging KS patients (Table 1). After 30 years of age, KS patients typically develop skin lesions (Meier et al., 2001; Hur et al., 2005; Nanda et al., 2006), although this has been subject of debate regarding the definite cause and definition of the skin defects observed in these patients (Hur et al., 2005; Cohen and Boyadjiev, 2006; Nanda et al., 2006). Transient patchy alopecia has also been reported in the follow-up of a patient initially described by Fryns et al. (Devriendt et al., 1999) and later confirmed in another case (Acar et al., 2010; Weaver et al., 2014). Interestingly, vascular calcification has been reported in only a few cases. In this regard, post-mortem examination of the youngest sibling originally described by Keutel uncovered calcification of pulmonary, coronary, hepatic, renal, meningeal and cerebral arteries (Cranenburg et al., 2011). Aortic calcification and intracranial-brain calcification have also been observed in a few other KS patients (Teebi et al., 1998; Ayyildiz et al., 2012; Bosemani et al., 2014; Weaver et al., 2014).

Most of the symptoms seen in KS are thought to be secondary to calcifications. Intracranial calcification could explain seizure, epilepsy, and even developmental delay. Tracheobronchial calcification could be responsible for dyspnea and cough by inducing trachea and bronchi stenoses and reducing respiratory tract elasticity. Finally, auricular calcification, sometimes leading to petrified ears of the pinnae or ligamentous calcification of the ossicles, could explain the hearing loss in KS patients, although frequent respiratory infections could also be a cause of the auditory defects. Finally, systemic hypertension found in most young patients and commonly controlled by standard antihypertensive medication is thought to be due to renal arterial microcalcification (Khosroshahi et al., 2014).

Some of the traits listed above for KS overlap with those found in other rare congenital or acquired diseases, including brachytelephalangic X-linked chondrodysplasia punctata (CDPX1; Maroteaux, 1989; Weaver et al., 2014), vitamin K-dependent gamma-glutamyl carboxylase (GGCX) mutation disorders (Tie et al., 2016), vitamin K deficiency embryopathy (Menger et al., 1997), warfarin embryopathy (Struwe et al., 1984; Kumar et al., 2012), or warfarin sodium therapy-induced complications (Moncada et al., 1992). Although it is likely that several of these diseases may involve functional alterations of the vitamin K-dependent activity of MGP, the exhaustive characterization of the reported KS patients (Table 1) now unequivocally recognizes KS as a separate disease that can be readily distinguished on the basis of morphological and clinical exams and further confirmed by genetic testing (*MGP* sequencing). This should help for better detection and care for KS patients whose prognosis is usually reasonably good. Recurrent cough and wheezing episodes as well as upper respiratory tract infections or seizures often lead to hospitalization and the incidental diagnosis of KS by chest radiograph. In fact, life expectancy of the patients mainly depends on the severity of their respiratory complications, which are usually treated symptomatically using antibiotics to prevent infections (Meier et al., 2001) and corticosteroids or inhalative bronchodilating drugs to improve the respiratory functions (Devriendt et al., 1999; Özdemir et al., 2006; Girit and Senol, 2019). However, such treatments are not always efficient (Meier et al., 2001; Cranenburg et al., 2011). Interestingly a vitamin K1 supplementation strategy was tried in a unique KS patient, where the presence of undercarboxylated MGP was characterized. However, this was found to be ineffective in increasing MGP carboxylation (Cranenburg et al., 2011). If patients have cosmetic complaints, plastic and reconstructive surgeries can be offered, e.g., nose grafting (Ciloglu et al., 2015). Although it has not been done so far, exploratory middle ear surgery could be considered for KS patients with conductive hearing loss (Acar et al., 2010).

## Identification of the Causal Gene

Matrix Gla protein is a vitamin K-dependent protein originally isolated from bovine bone (Price et al., 1983; Price and Williamson, 1985) and later found to be mainly expressed by chondrocytes and cardiovascular cells (Hale et al., 1988; Yao et al., 2007; Chiyoya et al., 2018). The human protein contains 84 residues and a complex molecular structure including a signal peptide required for its secretion into the ECM, a phosphorylation motif containing three serine residues located at the N-terminal end of the mature protein, separated from the remaining protein regions by a cleavage site (AXXF) followed by a γ-carboxylase recognition site and a Gla domain. It also contains a disulfide bridge between two cysteines and a C-terminal RR cleavage site (summarized in Figure 1B). The posttranslational modification of five glutamic acid (Glu) residues (see localization in Figure 1B) into γ-carboxyglutamic acid (Gla) residues is thought to be central to MGP function and requires the GGCX activity with vitamin K as a cofactor (Engelke et al., 1991). Although MGP is often considered to be fully active when phosphorylated and carboxylated, other forms (i.e., under-carboxylated or non-phosphorylated MGP) have been shown to have some clinical relevance, in particular for vascular calcification (Schurgers et al., 2010; Roumeliotis et al., 2019). MGP anti-mineralization action is currently not fully understood, although several mechanisms have already been proposed, e.g., its high affinity for calcium ions and calcium-phosphate crystals through the Gla residues would prevent mineral deposition or stimulate phagocytosis by macrophages, and its binding to BMP-2 would prevent the trans-differentiation of vascular smooth muscle cells into osteoblasts (see Roumeliotis et al., 2020 and references therein).

MGP structure has been remarkably conserved throughout evolution since its appearance 400 million years ago during the onset of cartilaginous structures, indicating that MGP plays a central function in vertebrates (Cancela et al., 2014). The human *MGP* gene was first sequenced in 1990 and localized in human chromosome 12p12.3-13.1 (Cancela et al., 1990). It was found to span four exons and it appears to be a single copy gene in all species identified to date. More recently, an additional exon was identified in single-pass cDNA sequences from human fetal brain precursor tissue (Cancela et al., 2014), bringing the total number of exons to five and adding 33 amino acid residues in frame with the previous protein. This new exon is specific to primates, is located between previously identified exons one and two and suffers alternative splicing in most tissues analyzed so far. Even though *MGP* gene was identified 30 years ago, its regulation is still unclear and much remains to be uncovered. So far, *MGP* was found to be regulated in human by retinoic acid (Kirfel et al., 1997) and FGF (Stheneur et al., 2003) and, more recently, by epigenetic mechanisms (Tiago et al., 2016; Tuo and Ye, 2017). In mouse, parathyroid hormone regulates *Mgp* expression through the transcription factors Sp and Runx2 (Suttamanatwong et al., 2009). In 1997, the development of a knockout (KO) mouse model for MGP provided crucial clues toward understanding the function of this enigmatic protein, until then without a significant physiological function (Luo et al., 1997). Its role as a physiological inhibitor of calcification was further consolidated in the next few years when it was identified as KS causal gene (Munroe et al., 1999). A genome search using homozygosity mapping provided evidence of the linkage of KS to the human chromosomal region 12p12.3-13.1 (maximum multipoint lod score, 4.06), where *MGP* gene is located, and the common traits displayed in both KS patients and the KO mice, including abnormal calcification of cartilage affecting auricles, nose, and respiratory tract, further supported the central role of MGP in this disease. Previously, Teebi et al. (1998) had provided evidence supporting the fact that KS was most likely due to an autosomal recessive inheritance. In the same year, Yagami et al. (1999) provided additional evidence for the function of MGP as a calcification inhibitor by overexpressing this protein in osteoblasts in the developing chick limbs resulting in a severe decrease in ECM mineralization and endochondral ossification. In the following two decades, eight different mutations in the *MGP* gene were identified in KS patients, all of them severely affecting protein structure and probably protein function (Figure 1B) and likely to be responsible for all or part of the phenotypes observed in KS patients (Figure 1C).

## Knockout and Transgenic Mouse Models to Understand the Pathologies of KS

Matrix Gla protein knockout (*Mgp*^–/–^) mice generated by Dr. Karsenty’s group is a widely used model for KS (Luo et al., 1997). These mice faithfully recapitulate most of the traits seen in the human patients, which include the widespread calcification of various cartilaginous tissues and associated skeletal anomalies. In addition, these mice display severe vascular calcification (Luo et al., 1997; Murshed et al., 2004; Leroux-Berger et al., 2011; Khavandgar et al., 2014), a trait that has only been reported in a limited number of KS patients (Cranenburg et al., 2011; Ayyildiz et al., 2012; Bosemani et al., 2014; Weaver et al., 2014). Unlike KS patients who are unaffected or mildly affected by vascular calcification, all *Mgp*^–/–^ mice die before two months of age due to complications (aortic rupture and hemorrhage) caused by severe vascular calcification (Luo et al., 1997).

The major cartilaginous tissues prematurely/abnormally mineralized in *Mgp*^–/–^ mice are trachea, nasal septum and growth plates, including the spheno*-*occipital synchondrosis (SOS) in the head (Marulanda et al., 2017). Interestingly, calcification of different cartilaginous tissues in MGP-deficient mice does not appear at the same time. The early calcium phosphate minerals in the nasal septum cartilage can be detected by histological methods by the end of the first week after birth (Marulanda et al., 2017). Most of the deposited minerals at this stage are amorphous calcium phosphate. The amount of minerals progressively increases over time and by the end of six weeks, septal cartilage appears to be fully mineralized. Almost simultaneously progressive deposition of minerals starts in the SOS at the cranial base, where mineral deposition occurs at the cartilaginous ECM of the synchondrosis leading to its premature closure (Marulanda et al., 2017). Abnormal calcification of the tracheal cartilage in *Mgp*^–/–^ mice is seen after the third week, while the growth plate cartilage in the long bones starts calcifying during the second month after birth (Marulanda et al., 2013).

As in KS patients, the premature and/or abnormal calcification of the cartilaginous tissues has profound effects on the development of the skeletal traits in *Mgp*^–/–^ mice. In-depth analyses of these traits in various mouse models have tremendously helped our understanding of the mechanisms causing general skeletal traits in KS, such as midface hypoplasia and shortening of some of the endochondral bones. Midface hypoplasia is commonly caused by the premature closure of the cranial sutures, sometimes in association with the premature closure of the SOS. However, in *Mgp*^–/–^ mice, the cranial sutures are not prematurely fused, but SOS are. It is possible that both SOS and nasal septum calcification contribute to the observed midface hypoplasia in both *Mgp*^–/–^ mice and in the KS patients.

Histological analyses reveal that the continuity of the prehypertrophic and proliferating cell layers in the growth plates of the endochondral bones of *Mgp*^–/–^ mice is disrupted by the premature deposition of minerals (Murshed et al., 2004). This phenotype may affect the growth of the long bones, although most of the *Mgp*^–/–^ mice die before the full manifestation of this phenotype. Following a transgenic approach, when vascular calcification in *Mgp*^–/–^ mice was prevented by vascular smooth muscle cell (VSMC)-specific restoration of *Mgp* expression, the early lethality was prevented. This tissue-specific rescue model provided a unique opportunity to examine the ultimate fate of the growth plates in the absence of MGP; the growth plates were almost completely lost with age in these mice due to abnormal deposition of minerals (Murshed et al., 2004). Note that unlike humans, mice do not show closure of the endochondral growth plates after puberty and maintain this cartilaginous tissue throughout the adulthood. As local expression of *Mgp* in the VSMCs prevents vascular calcification in *Mgp*^–/–^ mice, but not cartilage calcification, chondrocyte-specific expression of *Mgp* in *Mgp*^–/–^ mice prevents cartilage, but not vascular calcification (Marulanda et al., 2017). This observation suggests that MGP prevents ECM mineralization locally. Collectively, further analyses of the traits of these animal models may help us understanding some of the KS pathologies, such as the shortening of distal phalanges.

Several early studies suggested that MGP might inhibit signaling mediated by bone morphogenetic proteins (BMPs). It was proposed that the loss of MGP in the vascular tissues induces BMP signaling and trans-differentiation of VSMCs toward the chondrogenic/osteogenic lineage (Zebboudj et al., 2002). Although chondrocyte-like cells do appear in the heavily calcified arteries of *Mgp*^–/–^ mice, it is now evident that this alteration of cellular traits is secondary to mineral deposition (Leroux-Berger et al., 2011; Khavandgar et al., 2014). It is highly likely that MGP directly prevents mineral deposition on extracellular protein scaffold. This notion is supported by two *in vivo* observations – *firstly*, the initial mineral deposition in *Mgp*^–/–^ arteries occur before any upregulation of chondrogenic/osteogenic markers, and *secondly*, gene dose reduction of a mineral-scaffolding extracellular protein elastin in *Mgp*^–/–^ arteries significantly reduces the amount of deposited minerals (Khavandgar et al., 2014).

A direct role of MGP in the prevention of ECM mineralization will rely on specific features in its structure. Indeed, two post-translational modifications – the carboxylation of five glutamic acid residues (four in mice) by GGCX and phosphorylation of three N-terminal serine residues by yet unknown kinase(s) have been thought to be necessary for MGP’s anti-mineralization function. In a transgenic model, when MGP was overexpressed in bone, it resulted in a moderate bone mineralization defect. However, in a follow-up experiment when a mutated form of MGP lacking its glutamic acid residues undergoing carboxylation by GGCX was expressed in bone, it failed to cause any mineralization defect (Murshed et al., 2004). These experiments suggest a critical role of MGP’s conserved glutamic acid residues and their post-translational carboxylation in the skeletal tissues. Until now, no *in vivo* experiments have been performed to determine the role of the conserved serine residues in MGP in its anti-mineralization function. *In vitro* studies showed that an N-terminal human MGP peptide carrying the phosphorylated serine residues prevents mineral crystal formation (Schurgers et al., 2007; O’Young et al., 2011). More recently, Parashar et al. demonstrated that a similar mouse MGP peptide carrying the phosphorylated serine residues prevents mineral deposition in both cell culture and cell free models of elastin calcification (Parashar et al., 2021), while the non-phosphorylated control peptide did not. The ongoing studies using novel mutations in MGP’s conserved residues in our laboratories will provide critical information on how MGP prevents ectopic mineralization of soft tissues.

## Conclusion and Perspectives

Fifty years of research on Keutel syndrome have clarified both the phenotype and the genotype of the disease. Distinctive KS traits (e.g., abnormal cartilage calcification facial features and brachytelephalangism) can be easily identified through clinical observation and allow to positively diagnose the disease, while mutations in *MGP* gene identified through DNA sequencing will unequivocally confirm the diagnosis. In this regard, an effort should be made toward the sequencing of *MGP* gene in all the reported cases of KS – so far less than 30% of the KS patients were genotyped for *MGP* – to gain insights into a possible link between the severity of the disease and the type of mutation (Figure 1C). Although knockout mice have proven their usefulness to clearly associate a defective matrix Gla protein with the development of the disease, much remains to be discovered about MGP function in relation to Keutel syndrome. Currently, the underlying cause of midface hypoplasia in KS patients and Mgp-/- mice is not well-understood. It is possible that both nasal septum and SOS calcification contribute to this trait, however additional studies will be needed to determine the relative contributions of these two ectopic calcification events to the abnormal midface development in KS patients. Further, it is still unknown how MGP prevents ECM mineralization in cartilage and vascular tissues, and whether different post-translational modifications in MGP work in concert or independently to confer its anti-mineralization function. In this regard, the generation of new mouse models such as knock-in mice carrying targeted MGP mutations should provide interesting data on the function of the different protein domains/functional residues. Similar genetic approaches can be used to investigate the consequence of the mutations identified in KS patients. In addition, given the high level of conservation of MGP among species, the development of different animal models could contribute to shed further light into the mechanism of action of MGP and better understand its role in the development of this rare disease.

Although prognosis is good in most cases, the health conditions of KS patients are often far from normal with symptoms that can be a burden to them and their families. While reconstructive surgery, angiographic dilatation, antihypertensive and bronchodilating agents, and antibiotics can be used to treat some of these symptoms and improve patient condition, others such as developmental delay, seizures or hearing loss are only efficiently treated if tackled very early during development, before ectopic calcifications initiate in the different tissues. In this regard, gene therapy approaches to restore the normal expression of *MGP*, the causal gene for KS, may represent a pertinent solution for patients suffering from incapacitating symptoms.

## Author Contributions

All authors have contributed equally to the writing and review of this manuscript.

## Conflict of Interest

The authors declare that the research was conducted in the absence of any commercial or financial relationships that could be construed as a potential conflict of interest.
